# How Do Hospital Pharmacists Approach Substitution of Nanomedicines? Insights from a Qualitative Pilot Study and a Quantitative Market Research Analysis in Five European Countries

**DOI:** 10.3390/pharmaceutics13071010

**Published:** 2021-07-02

**Authors:** Natalia Sofia, Stefan Mühlebach, Umberto M. Musazzi, Rani Khatib, José Manuel Martinez Sesmero, Hans-Peter Lipp, Jacqueline Surugue, Tiziana Di Francesco, Beat Flühmann

**Affiliations:** 1 Drug Sciences and Toxicology Department, University of Basel, 4056 Basel, Switzerland; natalia.v.sofia@gmail.com; 2 Vifor Pharma Management Ltd., Global Headquarters, 8152 Glattbrugg, Switzerland; tiziana.difrancesco@viforpharma.com; 3 Division of Clinical Pharmacy & Epidemiology and Hospital Pharmacy, Department of Pharmaceutical Sciences, University of Basel, 4031 Basel, Switzerland; stefan.muehlebach@unibas.ch; 4 Department of Pharmaceutical Sciences, Università degli Studi di Milano, 20133 Milan, Italy; umberto.musazzi@unimi.it; 5 Medicines Management & Pharmacy Services and Cardiology Department, Leeds Teaching Hospitals NHS Trust, Leeds LS9 7TF, UK; R.Khatib@leeds.ac.uk; 6 Leeds Institute of Cardiovascular and Metabolic Medicine, University of Leeds, Leeds LS2 9JT, UK; 7 Hospital Pharmacy, San Carlos Clinical University Hospital, 28040 Madrid, Spain; jmmartinezs@gmail.com; 8 Hospital Pharmacy, University of Tübingen, 72076 Tübingen, Germany; Hans-Peter.Lipp@med.uni-tuebingen.de; 9 International Pharmaceutical Federation, 2517 The Hague, The Netherlands; jsurugue@gmail.com; 10 Hospital Pharmacy, Georges Renon General Hospital, 79000 Niort, France

**Keywords:** drug substitution, hospital formulary, hospital pharmacy, hybrid approval pathway, nanomedicines, nanosimilars, non-biologic complex drugs

## Abstract

We conducted research to assess hospital pharmacists’ familiarity with/interpretation of data requirements for the different regulatory approval frameworks and the impact of this on their approach to substitution in the formulary. The online questionnaire included a small molecule (acetylsalicylic acid—follow-ons approved via the generic pathway), two biologic drugs (insulin glargine and etanercept—follow-ons approved via the biosimilar pathway), a non-biologic complex drug (NBCD; glatiramer acetate—follow-ons approved via the hybrid pathway) and a nanomedicine, ferric carboxymaltose (no follow-ons approved as yet). The study was conducted in two phases: an initial qualitative pilot study with 30 participants, followed by a quantitative stage involving 201 pharmacists from five European countries. Most expected negligible safety/efficacy differences between reference and follow-on products. Head-to-head clinical data showing therapeutic equivalence as a prerequisite for reference product/follow-on substitution was perceived to be needed most for biologics (47%), followed by NBCDs (44%)/nanomedicines (39%) and small molecules (23%). Overall, 28% did not know the data requirements for follow-on approval via the hybrid pathway; 16% were familiar with this pathway, compared with 50% and 55% for the generic and biosimilar pathways, respectively. Overall, 19% of respondents thought the European Medicines Agency (EMA) was responsible for defining the substitutability of follow-ons. Education is required to increase hospital pharmacist’s knowledge of regulatory approval frameworks and their relevance to substitution practices.

## 1. Introduction

In recent years, the introduction and progress in nanotechnology has led to the development of a wide range of nano-based medicinal products, including innovative drugs termed nanomedicines [1,2]. Nanomedicines belong to a broad group of non-biologic complex drugs (NBCDs) [3] (Supplementary Table S1). The nanomaterial part of a nanomedicine formulation may be the active ingredient itself, a drug carrier, a novel excipient, or a drug complex/conjugate [4]. Nanomedicines include, but are not limited to, drug nanocrystals (e.g., olanzapine), liposomes (e.g., doxorubicin hydrochloride), polymeric drugs (e.g., glatiramer acetate), iron-polymer complexes (e.g., ferric carboxymaltose), virus-like particles (e.g., formalin inactivated hepatitis A virus) and virosomes (e.g., recombinant adenovirus expressing wildtype-p53) [5,6]. Between 1973 and 2015, liposomal agents were the most prevalent type of nanomedicine to be submitted for regulatory approval, followed by products containing nanocrystals, emulsions and iron-polymer complexes [4].

**Figure 1:**
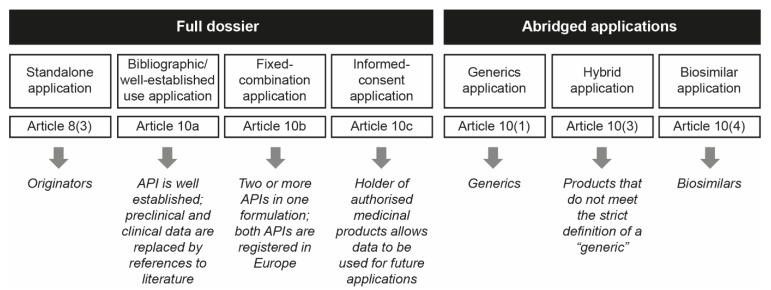
Schematic representation of the EMA approval pathways used for NBCD follow-on products (reproduced with permission from [12]). API = active pharmaceutical ingredient; EMA = European Medicines Agency; NBCD = non-biologic complex drug.

These chemically synthesized nanomedicinal products have a high level of complexity in their heterogeneous structures and composition that preclude full physicochemical characterization, posing a challenge for reproducible pharmaceutical development to ensure quality, safety and efficacy [1,7,8]. Due to the intrinsic features of nanomedicines, assessment of the appropriate regulatory evaluation process for follow-on nanomedicine products (or “nanosimilars”) versus the original reference products is still under debate [7,9]. As nanomedicines are not considered to be an independent category of medicinal products, no formal regulatory pathway has been established for the approval of follow-on nanomedicines. Most stakeholders are still unaware of the suggested data requirements for the approval of nanomedicine follow-ons and the assessment of their therapeutic equivalence versus their reference product [10]. In Europe, approval of follow-on medicinal products by the European Medicines Agency (EMA) and national regulatory authorities can be obtained via an abridged application process that involves one of three different pathways: a generic pathway, via Article 10(1), a hybrid pathway, via Article 10(3) or a biosimilar pathway, via Article 10(4) (Figure 1) [11,12].

The generic pathway is suitable for follow-on small-molecule products comprising the same substance, strength and pharmaceutical form as that of the reference product, with cross-referencing to the originator’s Common Technical Document modules 4 and 5 [13]. Moreover, these medicinal products need to present proof of bioequivalence, as demonstrated with appropriate bioavailability studies. Due to the small molecule’s unambiguously defined chemical structure, no additional non-clinical tests and/or clinical trials are required for these generics to confirm therapeutic equivalence.

The hybrid pathway is designed for follow-on medicinal products for which the strict definition of a “generic” is not met, i.e., bioequivalence cannot be shown, or the product differs from the reference product in its pharmaceutical formulation, indication, strength or administration route [14]. Hybrid medicines are those whose authorization depends both on the original data for the reference product and on new clinical trial data for the follow-on product [11,13].

The hybrid pathway can assess the “similarity” of a follow-on medicinal product compared with the reference product on a “weight of evidence approach”, which requires additional data from quality, non-clinical and human pharmacokinetic studies. This means that the hybrid pathway corresponds more closely to the biosimilar pathway than the generic pathway, where therapeutic equivalence is assessed based on physicochemical sameness and bioequivalence between the originator and its generic [10,13,15,16]. Some biological medicinal follow-on products are similar to a reference biological product, but do not meet the definition of a generic medicinal product [13]. This may be due to differences in the raw materials or manufacturing processes, related to the natural variability of the cell materials used and the fact that the complex nature of manufacturing biological products does not permit the molecular micro-heterogeneity to be perfectly replicated [2,17]. Here, a comparability exercise with suitable non-clinical assays and clinical trials is needed to assess similarity versus the originator product by following the biosimilar 10(4) regulatory pathway.

Although abridged applications for nanomedicine follow-ons can be submitted either via centralized or decentralized national procedures [10], to date, most nanomedicines available in Europe have been assessed by national authorities [12]. To guarantee consistency in the scientific evaluation of these products, legislative changes towards a mandatory centralized procedure have been requested by members of the European Parliament and expert organizations [18]. As of the end of 2018, 56% of nanomedicine follow-on applications had been approved via the generic pathway and 38% by the hybrid pathway, resulting in divergent evidence requirements across European countries [10]. Overall, in the last decade, the percentage of applications following the hybrid pathway for nanomedicine follow-ons has increased steadily over the number of generic ones [10]. Indeed, Klein et al. [10] reported that after 2015, these agents were exclusively approved via the hybrid or biosimilar pathways. This is most likely due to EMA guidance on regulation and data requirements for selected nanomedicine follow-ons, as well as the publication of scientific articles and EMA reflection papers acknowledging the failure of the “generic approach” for some “nanosimilar” products [7,16,19].

The implications of nanomedicine follow-on approvals in terms of handling and substituting these complex products in clinical practice remain unclear [15] and generally local (unharmonized) guidelines are followed. In many European countries, including Germany, Spain and the United Kingdom (UK), the use of international non-proprietary names (INNs) over the branded names is encouraged as a general cost-containment measure. This approach may facilitate the substitution of generics/follow-ons in the formulary, as they may be cheaper than the originator products. However, there may not be sufficient data to support an equivalent efficacy or safety of these follow-on products and the prescribing clinician may be unaware of the active substitution carried out by other stakeholders [6]. This strategy encompasses all follow-on medicinal products, irrespective of either their characteristics (small molecules, biologics or nanomedicines) or their approval pathway [20,21,22], even if prescribing physicians may counsel against substitutions under certain specific circumstances [23]. In France, the approach is even more strict, with the use of INNs compulsory except in very limited circumstances [24].

In daily clinical practice, hospital pharmacists have a central role in making substitution decisions [25]. Substitution of follow-on medicinal products approved via the generic pathway is usually straightforward, since with some exceptions, therapeutic equivalence can be assumed, and so national bodies have established clear rules on substitution and interchangeability. However, substitution policies and systems differ between countries. For example, in Italy, the situation differs to that in France, with only equivalent medicines (e.g., generics) included on the Italian Medicines Agency (AIFA) transparency list [26] having to be prescribed by INNs and therefore able to be automatically substituted in the hospital setting. This list includes mostly generics and their originators, but also other medicines with the same composition of active ingredients, pharmaceutical form, route of administration, unit number and dose, potentially including NBCDs. For biosimilars approved via the biosimilar pathway, additional clinical guidance for substitution practices is often required by pharmacists, especially as this pathway encompasses a broad spectrum of drug complexity, from insulin to monoclonal antibodies [27]. In light of this, although the AIFA recognizes the potential interchangeability of biosimilar originator and follow-on products, these are not included in the transparency list and, therefore, cannot be automatically substituted by hospital pharmacists [28]. Switching is allowed after the clinician’s review of the clinical history of the patient. The supply of medicines in Italian hospitals is built on a tendering system in which medicinal products (including biosimilars) are grouped based on their therapeutic equivalence. In this context, it is important that hospital pharmacists ensure that patients already being treated with a specific biosimilar can continue treatment with the same product, even if it is not the one that won the tender and/or was included in the hospital formulary.

**Figure 2:**
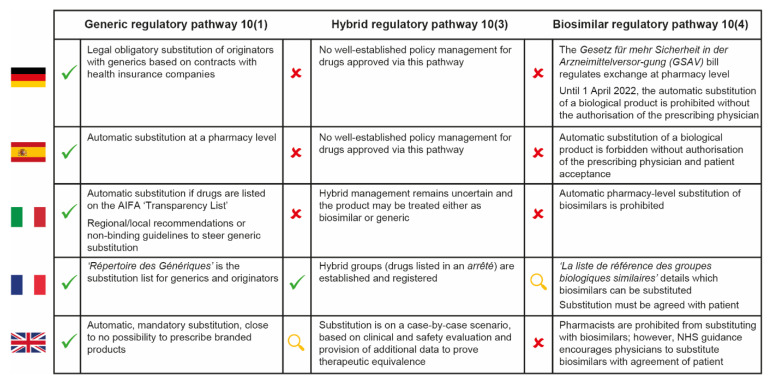
Cross-country comparison of hospital pharmacy substitution policies for generic, hybrid and biosimilar drugs in five European countries [29,30,31,32,33,34]. AIFA = Italian Medicines Agency; NHS = National Health Service.

In general, we expect hospital pharmacists to be less familiar with the substitution of “nanosimilars” approved via the hybrid pathway than with the substitution of generics or biosimilars, despite the recent increase in regulatory approvals for NBCDs. This reflects the lack of specific indications from the EMA and the heterogeneous approaches used across Europe for these complex medicinal products, as well as the lack of national rules for substitution (Figure 2) [29,30,31,32,33,34].

Here, we conducted research among a representative group of European hospital pharmacists to assess the hospital pharmacist’s interpretation of the three different abridged regulatory approval pathways for follow-on medicinal products, as a building block to understand pharmacist decision-making criteria for including follow-on products in the hospital formulary. We also evaluated their current approach for the selection and use of follow-on products, focusing on their needs and understanding relating to data requirements.

## 2. Materials and Methods

This market research study consisted of two key phases: an initial qualitative pilot study with a limited number of participants (*n* = 30), which was followed by a quantitative data collection stage (*n* = 201). Both phases included a similar number of pharmacists from each of the five participating European countries: France, Germany, Italy, Spain and the UK. The research was carried out between 24 January 2020 and 13 March 2020.

### 2.1. Qualitative Pilot Study

The aim of the initial qualitative pilot study was to provide a high-level view of the current substitution landscape and to help guide the development of the questions for inclusion in the subsequent quantitative data collection phase. It was also used to check for understanding of the questionnaire to be used in the subsequent quantitative study, verify the accuracy and completeness of the information relating to the research topic, check for possible redundancy among the questions, validate the data collection method and target the most relevant participant sample. Data from the qualitative study were used to supplement the results from the quantitative study, where appropriate.

The initial pilot study consisted of semi-structured, 60 min telephone interviews with a total of 30 pharmacists selected from hospitals in the five participating European countries (France, Germany, Italy, Spain and the UK). The interviews were conducted by telephone with screen-sharing and included questions covering the following topics: substitutability perception and differentiation, current practices and guidelines for substitution, hybrid drugs and approval pathway, and key decision-makers. The selection of the hospital pharmacists was based on specific, predefined screening criteria, to ensure that pharmacists with adequate knowledge and experience in the hospital setting and with influence on substitution guidelines, protocols and purchasing decisions were targeted (Table 1). No introduction to the concept of nanomedicines or “nanosimilars” was provided to ensure that all responses received were spontaneous/unbiased.

### 2.2. Quantitative Market Research

The quantitative market research involved a 15 min, structured online questionnaire for completion by hospital pharmacists in France, Germany, Italy, Spain and the UK. Approximately 40 pharmacists from different hospitals in each participating country were targeted, selected based on the same criteria as for the qualitative pilot study (Table 1). The questionnaire (supplementary Appendix) was used in the relevant local language (English, French, German, Italian and Spanish). The questions aimed to evaluate the perceptions of differences between reference and follow-on products (including nanomedicines) approved via the three different abridged European regulatory approval pathways, the extent to which hospital pharmacists were familiar with the three pathways, as well as their knowledge of the data requirements for approval of follow-on medicinal products. Beliefs relating to responsibility for defining substitutability, the main inclusion criteria for drugs approved via the hybrid regulatory pathway to be added to the hospital formulary, the key stakeholders responsible for decisions relating to the substitution status of hospital drugs, and opinions on the pathway most suitable for approval of nanomedicines were also addressed.

To evaluate perceptions of differences between reference and follow-on products, and data requirements for substitution of medicinal products in the formulary/switching in a given patient, the questionnaire provided Aspirin^®^ (acetylsalicylic acid) as an example of a branded small-molecule originator product where follow-on products have been approved via the generic regulatory pathway. Lantus^®^ (insulin glargine) and Enbrel^®^ (etanercept) were used as examples of biologics that have been approved following the biosimilar pathway. Copaxone^®^ (glatiramer acetate) was used as an example of a branded complex originator product (NBCD) where commercialized drug copies have been approved via the hybrid regulatory pathway [10]. Pharmacists were also questioned on expected differences between Ferinject^®^ (ferric carboxymaltose) and its hypothetical follow-ons (none were available in these markets at the time of study) as an example of a nanomedicine. In the results section, the use of brand names has been avoided, with data reported in general for the class of agents represented by each particular drug, where possible. Pharmacists’ familiarity with approval pathways was rated on a scale of 0–10, where 0 = not at all familiar and 10 = very familiar.

The questionnaire was designed by Exevia GmbH (Nürnberg, Germany). Exevia GmbH also supported with the interview process and subsequently performed the data analysis. Approval of the study questionnaire by English, French, German, Italian and Spanish centres was coordinated by the market research department of Vifor Pharma Group.

## 3. Results

Overall, 30 hospital pharmacists took part in the initial qualitative pilot study; this comprised six pharmacists from each of the five participating European countries (France, Germany, Italy, Spain and the UK). A total of 201 hospital pharmacists participated in the subsequent quantitative research study, comprising 40 pharmacists from each of France, Germany, Italy and Spain, and 41 from the UK (Table 2).

### 3.1. Perception of Drug Substitution and Use of Follow-On Products

**Figure 3:**
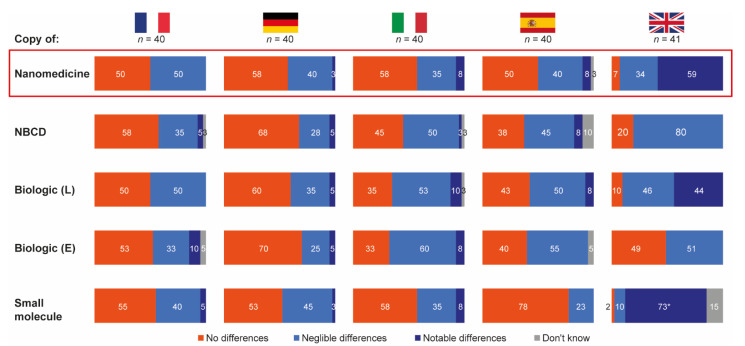
Expected differences in safety and/or efficacy between selected reference products and their follow-ons. Source question: For each of the following branded drugs, please imagine that a copy was to become available in your country. To what extent would you expect each copy to be identical, in terms of safety and/or efficacy outcomes to its originator? (Choose one option only). Data in bars are percentages of pharmacists choosing that option. UK pharmacists state the differing posology for small molecules as a reason for notable but not necessarily relevant differences. E = Enbrel^®^ (etanercept); L = Lantus^®^ (insulin glargine); NBCD = non-biologic complex drug.

The vast majority of hospital pharmacists from France, Germany, Italy and Spain stated that they expected negligible or no differences between reference products and their follow-ons in terms of safety and/or efficacy outcomes for all drug types included in the research. In contrast, a considerable proportion of pharmacists from the UK expected notable differences between reference and follow-on products for biologics (44%), nanomedicines (59%) and small molecules (73%) (Figure 3).

In general, the qualitative research suggested that pharmacists viewed the use of follow-on products positively due to the perceived economic benefits, improved drug access for patients and fewer supply issues. The pharmacists interviewed also noted a long-standing acceptance of equivalence between generics and reference products and stated that biosimilars are becoming more accepted as experience with this class of drugs increases.

### 3.2. Data Requirements for Substitution in the Hospital Formulary

**Figure 4:**
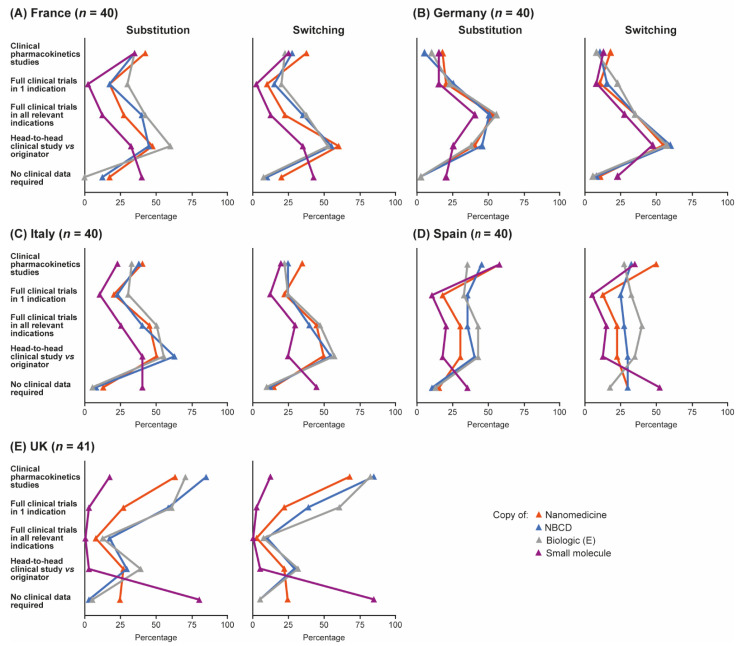
Data pharmacists would be required in order to be able to substitute a reference product for a follow-on in the hospital formulary, or to be able to switch prescriptions on a given patient. Data are shown by country: (**A**) France, (**B**) Germany, (**C**) Italy, (**D**) Spain and (**E**) UK. Source question: For each of the following branded drugs, please again imagine that a copy was to become available in your country. Please now imagine that your institution was to include BOTH the originator and the copy in the formulary (even if this may not be standard practice). What clinical data would your institution require in order to substitute the originator for the copy in your formulary/demand in order to be able to switch prescriptions freely between the branded originator and the copy, even within the same patient? (Choose as many options as required.) E = Enbrel^®^ (etanercept), NBCD = non-biologic complex drug.

Data requirements for substituting reference products with follow-ons in the hospital formulary and for switching in a given patient were broadly similar across countries, but with some variation (Figure 4A–E). In France, Italy and Germany, more pharmacists stated that they would require head-to-head clinical trial data for switching the originator with the follow-on within a given patient compared with the number stating that they would require this type of data for substituting within the formulary. In Spain, slightly more pharmacists responded that they would demand a clinical trial and pharmacokinetic data for inclusion of a follow-on in the hospital formulary compared with switching in a given patient, particularly for some small-molecule generics such as acetylsalicylic acid and for nanomedicines. UK pharmacists’ requirements for clinical data were the same regardless of whether substituting within the formulary or switching in a patient; in general, data demands were lower in the UK than in the other four countries but varied considerably by product. Overall, the need for head-to-head clinical data to permit substitution of a reference product with a follow-on was perceived to be greatest for the biologic Enbrel^®^; 47% of pharmacists thought head-to-head data would be required to permit substitution of this agent with a follow-on in the formulary. Similar proportions of pharmacists thought that head-to-head data would be needed to substitute follow-ons for either the NBCD Copaxone^®^ (44%) or the nanomedicine Ferinject^®^ (39%); fewest thought that head-to-head data were needed for small-molecule generics such as Aspirin^®^ (23%).

In contrast, qualitative insights suggested that while clinical data may be expected by pharmacists, this is often a formality, and in practice, data are not reviewed in detail at the hospital/pharmacist level, and guidance and review is expected from regulators.

### 3.3. Knowledge of Data Requirements for Follow-On Products

**Figure 5:**
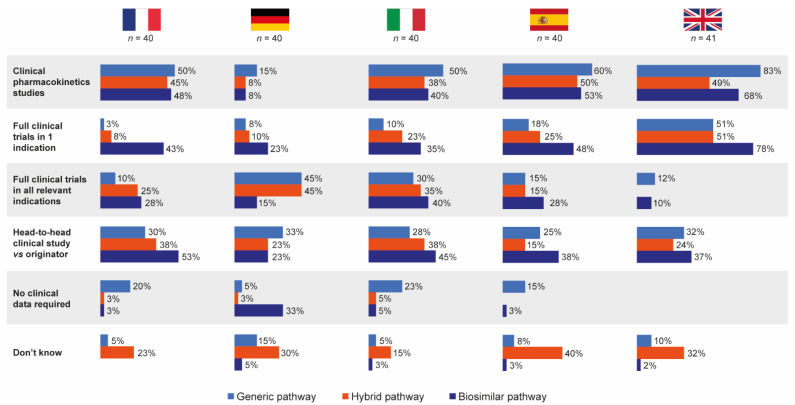
Pharmacist self-perceived knowledge of data required for approval by each European regulatory approval pathway (generic, hybrid and biosimilar). Source question: Based on your knowledge, what clinical data do copy drug manufacturers have to provide as part of each of the following EMA approval application pathways? (Choose as many options as required.) EMA = European Medicines Agency.

Hospital pharmacists completing the questionnaire did not significantly differentiate between the generic, biosimilar and hybrid EMA approval pathways in terms of the types of clinical data they thought were required by each for follow-on applications (Figure 5).

Overall, head-to-head clinical trial data for originator versus follow-on were perceived to be required most for products approved via the biosimilar pathway (39%), with 29% and 27% believing head-to-head data were needed for products approved via the generic and hybrid pathways, respectively. Responses were generally similar by country. In total, 28% of pharmacists stated that they did not know the data requirements for the hybrid pathway, compared with 8% and 2% for the generic and biosimilar pathways, respectively.

### 3.4. Awareness of European Regulatory Approval Pathways

**Figure 6:**
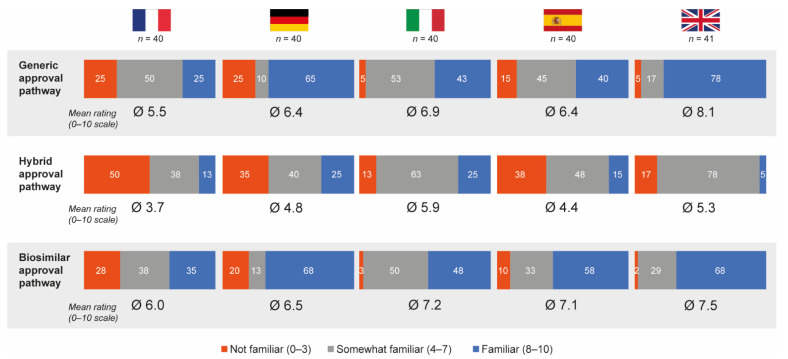
Pharmacist self-perceived familiarity with generic, hybrid and biosimilar regulatory approval pathways. Data in bars are percentages. Source question: How familiar are you personally with the following approval application pathways for drug copies from the EMA? Please indicate your level of familiarity on a scale from 0 (not familiar at all) to 10 (very familiar). EMA = European Medicines Agency.

Overall, 50% and 55% of hospital pharmacists who completed the questionnaire felt that they were familiar (score of 8–10 on the 10-point scale) with the generic and biosimilar approval pathways, respectively; only 16% were familiar with the hybrid approval pathway. Mean familiarity ratings were 6.6 for generic approval, 6.9 for biosimilar approval and 4.8 for hybrid approval. French pharmacists were the least familiar with all approval pathways (mean ratings 5.5, 6.0 and 3.7 for the generic, biosimilar and hybrid approval pathways, respectively) and Italian pharmacists had the highest average familiarity ratings for the hybrid pathway (mean rating 5.9) (Figure 6). With the exception of pharmacists from Germany, most pharmacists believed nanomedicine follow-ons should be approved via either the hybrid (44%) or biosimilar (39%) pathways.

Hospital pharmacists participating in the qualitative pilot study generally had a poor knowledge or understanding of the hybrid regulatory pathway. The exception was pharmacists from Italy, who reported partial knowledge on the regulations and the differences among the drugs approved via the hybrid pathway, as well as a basic understanding of the unique features of nanomedicines and the differentiation in bioequivalence and therapeutic substitution in contrast with generics.

### 3.5. Responsibility for Defining Substitutability

**Figure 7:**
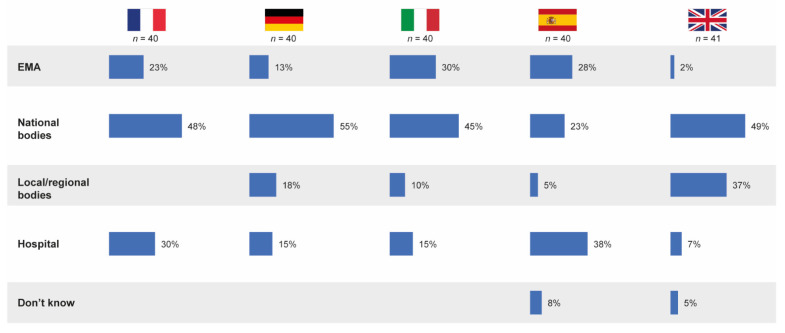
Stakeholders believed by pharmacists to be relevant in the definition of substitutability of follow-on products in the hospital formulary. Source question: Thinking of drug copies, who defines/would define whether it is substitutable with its originator? (Choose one option only; % denotes % of pharmacists choosing that option.) EMA = European Medicines Agency.

Overall, almost one in five pharmacists (19%) believed the EMA to be responsible for defining whether a follow-on medicinal product is substitutable with its reference product in the hospital formulary, irrespective of its approval pathway. However, 44% of pharmacists believed national bodies to be responsible for defining substitution rules and 21% thought the hospital was responsible. In most participating countries, the highest proportion of hospital pharmacists believed that national bodies defined whether a follow-on is substitutable; however, in Spain, 38% of pharmacists believed this decision was taken by the hospital (Figure 7).

In the qualitative pilot study, there was a strong belief that substitution of an originator product with a follow-on in the formulary is feasible, as long as EMA regulators approve the follow-on medicine. However, in most cases, pharmacists stated that they rely on national bodies to have scrutinized data and to give guidance on drug differences.

### 3.6. Inclusion of Hybrid Pathway-Approved Follow-Ons in the Hospital Formulary: Who Decides?

According to the hospital pharmacists responding to the questionnaire, the most common stakeholders thought to be involved in the decision to include a follow-on drug approved by the hybrid pathway in the formulary were the hospital drugs/medicines committee (65%), national regulatory bodies (43%) and the head hospital pharmacists (41%). This was generally consistent across countries, although prescribing clinicians were reported to have a role in the decision by 65% of pharmacists in France, and regional drugs/medicines committees by 76% in the UK (Supplementary Figure S1).

Qualitative insights confirmed that pharmacists regarded committees and regional/local bodies to have greater importance than individuals at a hospital level in the substitution of biosimilars in the formulary, whereas substitution of generics could mostly be initiated by the pharmacist.

## 4. Discussion

This quantitative market research, supplemented by qualitative data from an initial pilot study, demonstrates that the vast majority of hospital pharmacists interviewed from France, Germany, Spain and Italy expect no or negligible differences between nanomedicines and their follow-on products. Pharmacists in the UK were an exception, with over 50% stating that they expected notable differences between nanomedicines and their follow-on products. The qualitative research suggested that this may be due to perceptions around the complexity of nanomedicines and structural differences between originator and their follow-on products. Nonetheless, this research also suggested that pharmacists did not consider any differences to be significant or relevant to outcomes.

Irrespective of their country of practice, many of the pharmacists interviewed stated that head-to-head clinical trial data confirming therapeutic equivalence and, therefore, the interchangeability of follow-on and originator products would be required to permit substitution of these products in the hospital formulary, especially when considering biosimilars and NBCDs, including nanomedicines. This is in line with recommendations from a recent publication from a group of regulators and hospital pharmacists, which highlighted the need for robust clinical assessment of comparability and/or therapeutic bioequivalence of NBCDs and their follow-ons, rather than the current focus on preclinical and/or physicochemical characterization [6]. Many pharmacists also believed that clinical studies are conducted to specifically demonstrate therapeutic equivalence of originator and follow-on products, and wrongly assumed that such data are required by the EMA as part of the associated approval processes. There was also a strong perception that the EMA provides a therapeutic equivalence rating to define substitutability of follow-on products and that suitability for substitution is therefore implied along with regulatory approval. There is, therefore, a discrepancy between what data pharmacists may need/what they assume is required as part of the regulatory process and what is actually done to assess therapeutic equivalence and substitutability of originator and follow-on products.

There was a lack of knowledge of data requirements for approval of follow-ons via each of the European regulatory approval pathways, but especially for the hybrid pathway. There was also little differentiation between the pathways in terms of the data they understand to be required for each. As a result, a discrepancy exists between the level of data required for product approval by regulatory authorities and the data pharmacists believe should be provided to permit substitution of nanomedicines and their follow-ons in the hospital formulary. This may lead to the perception that there are no differences between nanomedicines and their follow-on products. However, despite pharmacists stating that they would require additional clinical data for substitution of these products in the formulary, this may be a formality, as there was an expectation that health authorities (both national regulatory bodies and the EMA) scrutinize the data and provide guidance on the use of follow-on products (almost 20% believed that the EMA was responsible for defining whether a follow-on product was substitutable with the reference product).

Consistent with this and irrespective of country, hospital pharmacists were considerably less familiar with the hybrid approval pathway than both the generic and biosimilar approval pathways. Overall, around half of the pharmacists surveyed felt they were familiar with the generic and biosimilar pathways, while only 16% were familiar with the hybrid pathway. Familiarity was likely higher with the generic pathway as it is well-established, based on decades of research and experience. Pharmacist experience with the biosimilar pathway is increasing as more biosimilar therapies become available. In contrast, the hybrid pathway is relatively unknown. Italian pharmacists had the highest average familiarity ratings for the hybrid pathway, which may be due to a recent court case in Italy regarding substitution of glatiramer acetate, a product that was approved via the hybrid pathway, bringing this issue into the public eye [35].

Overall, this research confirmed the central role of hospital pharmacists in the substitution of follow-on products approved by the hybrid pathway in European hospital formularies. This highlights a need for increased awareness and support to ensure that nanomedicine follow-ons are used appropriately and to address non-homogeneous practices across countries. Notably, a high proportion of pharmacists felt that it was within their remit to substitute “nanosimilars” without consulting a prescribing physician. Pharmaceutical experience-based decision-making is therefore strongly encouraged [15] and a tool has previously been developed to assist pharmacists with rational decision-making relating to the inclusion of nanomedicines in the hospital formulary [2]. This includes specific criteria to evaluate the substitutability or interchangeability of originator medicinal products and their follow-ons. Educational initiatives to improve pharmacists’ understanding of nanomedicines in terms of structure and activity profile would also be of value to support substitution decisions.

Establishing an appropriate regulatory framework for nanomedicines and their follow-ons and improving the consistency of prescribing practices in Europe would help to harmonize the practical use of these complex products and ensure their safety and efficacy for the patient [10,15,25]. Currently, national authorities, clinicians and pharmacists are required to translate the decision of regulators to determine prescribing practices, leading to inconsistent practices between countries, as shown in the current study. Additionally, hospital pharmacist scientific societies should issue clear statements regarding the interchangeability and substitution of nanomedicines and “nanosimilars”. For example, current guidance for pharmacists on the substitution of biologics and biosimilars [36] could be extended to include the selection and substitution of nanomedicines and “nanosimilars” [25].

Strengths of this study include the large sample size for the quantitative research, the inclusion of pharmacists from five different European countries and the use of an initial qualitative pilot study to guide the development of the questions for quantitative data collection. A limitation of this study is that it included only five European countries, thus the applicability of these results to other countries in Europe is unknown. Additionally, hospital pharmacists agreeing to participate in this research may have been more likely to have an interest in the subject, introducing potential selection bias. Moreover, the drugs selected as stimulus for the research might not permit generalization of the results to all the drugs of that type available in the market. This was also a descriptive study. Due to the relatively low number of hospital pharmacists from each country who were likely to meet the study eligibility criteria, no statistical analyses of the results were planned/performed, including no analysis of any between-country differences. Nevertheless, it must be noted that the quantitative study included a total of 201 hospital pharmacists, who were evenly distributed between the five participating countries (approximately 40 per country), ensuring a high proportion of experienced pharmacists from these regions were included. Therefore, we believe that these data might be representative of the views of hospital pharmacists across these countries as a whole.

## 5. Conclusions

In addition to requiring knowledge of the drug pricing system, local insurance reimbursement and health economics, hospital pharmacists have a central and unique role in both hospital formulary drug selection and substitution processes. Given the complexity surrounding the evaluation of drugs approved via the hybrid pathway, we encourage national and international regulatory agencies to issue clear guidelines on how hospital pharmacists should handle the dispatch of these complex drugs, mimicking, for example, the rating system on therapeutic equivalence already reported in the United States Food and Drug Administration Orange/Purple Books. At the same time, continuing education programmes should be accessible to hospital pharmacists to increase awareness and knowledge of nanomedicines and of their regulatory approval framework, focusing on data requirements for establishing therapeutic equivalence of these complex drugs and their follow-ons, as currently a knowledge gap appears to be present in this class of key stakeholders.

## Figures and Tables

**Table 1:** Screening criteria for selection of participating hospital pharmacists.

Type of institution	Hospital/clinic
Role in the institution	Pharmacist, pharmacist purchasing manager
Years of experience	3–30
Age	30–60 years
Extent of involvement in procurement and listing of off-patent pharmaceuticals	Be sole decision-maker for purchasing and listing Be one of the main decision-makers for purchasing and listing Have some influence on purchasing and listing, but not a main decision-maker
Extent of involvement in setting hospital guidelines and protocols	Be solely responsible for setting guidelines and protocols for drug substitution Be one of the main decision-makers for setting guidelines and protocols for drug substitution Have some influence on setting guidelines and protocols for drug substitution, but not a main decision-maker

**Table 2:** Socio-demographic characteristics of participating hospital pharmacists.

**Type of institution, *n* (%)**	
▪ Hospital	201 (100)
**Role in the institution, *n* (%)**	
▪ Pharmacist ▪ Formulary pharmacist ▪ Head pharmacist ▪ Pharmacist purchasing manager	88 (44) 11 (5) 83 (41) 19 (9)
**Mean (standard deviation) years of experience**	17.4 (6.4)
**Mean (standard deviation) age, years**	45.6 (6.9)
**Extent of involvement in procurement and listing of off-patent pharmaceuticals, *n* (%)**	
▪ Sole decision-maker ▪ One of the main decision-makers ▪ Some influence, but do not belong to the main decision-makers	37 (18) 142 (71) 22 (11)
**Extent of involvement in setting hospital guidelines and protocols for drug substitution, *n* (%)**	
▪ Solely responsible ▪ One of the main decision-makers ▪ Some influence, but do not belong to the main decision-makers	23 (11) 156 (78) 22 (11)

## Data Availability

The data presented in this study are available on request from the corresponding author. The data are not publicly available as they contain information that could compromise the privacy of research participants.

## Supplementary Materials

The following are available online at https://www.mdpi.com/article/10.3390/pharmaceutics13071010/s1, Table S1: Glossary of terms [1,2,3], Appendix: The hospital pharmacist market research questionnaire, and Figure S1: Stakeholders believed by pharmacists to be relevant in the decision to include a hybrid pathway-approved follow-on drug in the hospital formulary.
