# Supplementary material for: How Do Hospital Pharmacists Approach Substitution of Nanomedicines? Insights from a Qualitative Pilot Study and a Quantitative Market Research Analysis in Five European Countries
Source: Pharmaceutics. 2021 Jul 2;13(7):1010. doi: 10.3390/pharmaceutics13071010 (PMC8309126; doi:10.3390/pharmaceutics13071010)
Supplement: Supplementary file 1 [file pharmaceutics-13-01010-s001.zip › pharmaceutics-1244100-supplementary.pdf]

pharmaceutics

Supplementary Material: How Do Hospital Pharmacists Approach Substitution of Nanomedicines? Insights from a Qualitative Pilot Study and a Quantitative Market Research Analysis in Five European Countries

Natalia Sofia, Stefan Mühlebach, Umberto M. Musazzi, Rani Khatib, José Manuel Martinez Sesmero, Hans-Peter Lipp, Jacqueline Surugue, Tiziana Di Francesco and Beat Flühmann

Table S1. Glossary of terms [1–3].

Nanomedicine	A medicinal product developed and manufactured using nanomaterials and/or nanotechnology, often comprising multiple biological and non-biological structures
"Nanosimilar"	A follow-on product of a reference nanomedicine
Non-biologic complex drug (NBCD)	A non-biological medicinal product that consists of different (closely related) and often nanoparticulate structures that cannot be isolated, fully quantitated, characterised and/or described by physicochemical analytical means. As it is unknown which structural elements might affect therapeutic performance of an NBCD, their composition, quality, and *in vivo* performance are highly dependent on having a robust manufacturing process for both the active ingredient and the formulation
Interchangeability	Therapeutic equivalence of two different medicinal products enables the products to be interchanged. At a population level, this means that both products can be used for the treatment of the same condition in the same population. At an individual level, it means that the products can be alternated or switched as they are expected to have the same clinical effect
Substitution (automatic)	The pharmacy practice of dispensing one medicinal product instead of another equivalent and interchangeable medicinal product without consulting with the prescriber or gaining their consent
Switching	The practice of a prescriber/treating physician exchanging one medicinal product for another with the same therapeutic intent, during the course of a patient's treatment

References

1. Astier, A.; Barton Pai, A.; Bissig, M.; Crommelin, D.J.A.; Flühmann, B.; Hecq, J.D.; Knoeff, J.; Lipp, H.P.; Morell-Baladrón, A.; Mühlebach, S. How to select a nanosimilar. *Ann. N Y Acad. Sci.* **2017**, *1407*, 50–62.
2. European Medicines Agency (EMA). European Commission. Biosimilars in the EU. Information for Healthcare Professionals. 2021. Available on: https://www.ema.europa.eu/en/documents/leaflet/biosimilars-eu-information-guide-healthcare-professionals_en.pdf (accessed on 28 April 2021).
3. Crommelin, D.J.; de Vlieger, J.S.; Weinstein, V.; Mühlebach, S.; Shah, V.P.; Schellekens, H. Different pharmaceutical products need similar terminology. *AAPS J.* **2014**, *16*, 11–14.

Appendix

The hospital pharmacist market research questionnaire.

Substitution Practices

0. Introduction

Dear Sir/Madam/Doctor,

Thank you very much for your interest in this online survey conducted by Exevia GmbH, an independent market research and consulting company based in Nuremberg, Germany.

Survey Background

The project is about **regulatory approval pathways and drug substitution**, the purpose of which is to **gain a better understanding of how drug copies are approved and managed in your country.**

You will need about ***15*** minutes to complete the survey. For your time and effort, you will receive compensation as agreed.

This study is sponsored by a pharmaceutical company but is strictly non-promotional and no personal information will be passed to the company. The name of the sponsoring company will be revealed at the end of the interview so as to not bias any answers given.

Privacy and Confidentiality

Exevia GmbH is fully accredited and adheres to all professional and ethical codes of conduct *(namely ESOMAR and EphMrA guidelines)* and data protection legislation *(namely the EU General Data Protection Legislation).* As a participant in market research, you have a number of rights should you be willing and eligible to take part:

- The right to anonymity (no names or personal information will be passed on to the company sponsoring the research nor any other third party)
- The right to complete confidentiality (no comments will be attributed to a particular person in any subsequent report)
- The right to withdraw from the research at any time

For more information about your rights, please see our privacy notice, which is available at: www.exevia.com/en/data-privacy.html.

By agreeing to participate in this survey, you also agree to maintain the confidentiality of any proprietary materials that may be shown in the survey (usage, copying and disclosure of such materials is prohibited without consent).

Adverse Events

We are required to pass on to our client details of adverse events/product complaints pertaining to their products that are mentioned during the interview. If this happens, we will need to collect details and report the event, even if it has already been reported by you directly to the company or the regulatory authorities. You will be asked whether you consent to us passing your details to the client company's drug safety department for their follow-up, but you may choose to remain anonymous. This will have no impact on the confidentiality and anonymity associated with the interview itself and any other answers given.

Do you agree to continue under these terms?

- ❑ Yes → *Continue*
- ❑ No → *Thank and terminate*

At the beginning, we have a few questions to help make sure that we achieve the right mix of respondents.

1. Screener

S1. Which setting do you mainly work in?

One answer possible

- ❑ Mainly hospital/clinic → *Continue*
- ❑ Mainly retail pharmacy → *Thank and terminate*

S2. What is your role in your institution?

One answer possible

- ❑ Pharmacist → *Continue*
- ❑ Head pharmacist → *Continue*
- ❑ Pharmacist purchasing manager → *Continue*
- ❑ Other → *Thank and terminate*

S3. How many years have you been working as a pharmacist for?

Numerical answer, range 0 to 99

_______ years → *End if <3 years and if >30 years*

S4. Please state your age.

Numerical answer, range 0 to 99

_______ years old → *End if >60 years and if <30 years*

S5. To what extent are you involved in making purchasing and listing decisions for **off-patent pharmaceuticals such as generics and biosimilars (copy drugs)** in your institution?

- ❑ I am the **sole decision-maker** for purchasing and listing
- ❑ I am **one of the main decision-makers** for purchasing and listing
- ❑ I have some **influence on purchasing and listing**, but I do not belong to the main decision-makers
- ❑ **I am not involved** in purchasing and listing decisions → *Thank and terminate*

S6. To what extent are you involved in **setting guidelines and protocols for substitution of originator pharmaceuticals** with copies?

- ❑ I am **solely responsible** for setting guidelines and protocols for drug substitution

- ❑ I am **one of the main decision-makers** for setting guidelines and protocols for drug substitution
- ❑ I have some **influence on setting guidelines and protocols for drug substitution**, but I do not belong to the main decision-makers
- ❑ I have **no influence and am not involved** in setting guidelines and protocols for drug substitution → *Thank and terminate*

S7. For which of the following drugs are you personally involved in purchasing and listing decisions in your institution?

Randomise list

Multiple answers possible

- ❑ Diabetes drugs, including insulin
- ❑ Blood management drugs, including IV iron
- ❑ Antibiotics
- ❑ Neurology drugs, including anticonvulsants
- ❑ Asthma/COPD drugs, including inhaled corticosteroids
- ❑ Pain medications, including morphine
- ❑ Rheumatology drugs, including TNF blockers
- ❑ Immunology drugs, including immunomodulators
- ❑ Cardiology drugs, including antihypertensives
- ❑ Oncology drugs, including monoclonal antibodies

END if less than 5 drug areas selected–these MUST include:

- *"Blood management drugs, including IV iron"*

Thank you, you are now entering the main survey.

In this survey, we will be asking about drug substitution.

In this context, we are referring to drugs approved as copies of originators/reference listed drugs. These could be drugs of any type.

2. Perception of Substitutability and Criteria for Assessment

1. For each of the following branded drugs, please imagine that a copy was to become available in your country.

To what extent would you expect each copy to be **identical, in terms of outcomes**, to its originator?

Please drag and drop each drug copy into the relevant category below.

Drag and drop exercise

Randomise order of items

- Copy of the IV iron, iron carboxymaltose (Ferinject®)
- Copy of the immunomodulator, glatiramer acetate (Copaxone®)
- Copy of the insulin, insulin glargin (Lantus®)
- Copy of the TNF inhibitor, etanercept (Enbrel®)

- Copy of the painkiller, acetylsalicylic acid (Aspirin®)

Drag and drop answer options

- ❑ No differences in outcomes possible *vs* branded originator
- ❑ Negligible differences in outcomes possible *vs* branded originator
- ❑ Notable differences in outcomes possible *vs* branded originator
- ❑ Don't know

2. For each of the following branded drugs, please again imagine that a copy was to become available in your country.

What clinical data would **your institution** require in order to **substitute the originator for the copy in your <u>formulary</u>**?

Please select all the data that you/your institution would require.

One screen per drug

Randomise order and keep this order for remaining questions in this section

- Copy of the IV iron, iron carboxymaltose (Ferinject®)
- Copy of the immunomodulator, glatiramer acetate (Copaxone®)
- Copy of the TNF inhibitor, etanercept (Enbrel®)
- Copy of the painkiller, acetylsalicylic acid (Aspirin®)

Multiple answers possible

- ❑ Clinical pharmacokinetics studies
- ❑ Full clinical trials in 1 indication
- ❑ Full clinical trials in all relevant indications
- ❑ Head-to-head clinical study *vs* originator

Single answer

- ❑ No clinical data required

3. Please now imagine that your institution was to **include BOTH the originator and the copy in the formulary (even if this may not be standard practice)**.

What clinical data would your institution demand in order to be able to **<u>switch prescriptions freely</u> between the branded originator and the copy**, even **within the <u>same patient</u>**?

Please select all the data that you/your institution would require.

One screen per drug, order as in Q2

- Copy of the IV iron, iron carboxymaltose (Ferinject®)
- Copy of the immunomodulator, glatiramer acetate (Copaxone®)
- Copy of the TNF inhibitor, etanercept (Enbrel®)
- Copy of the painkiller, acetylsalicylic acid (Aspirin®)

Multiple answers possible

- ❑ Clinical pharmacokinetics studies
- ❑ Full clinical trials in 1 indication

- ❑ Full clinical trials in all relevant indications
- ❑ Head-to-head clinical study vs originator

Single answers:

- ❑ No clinical data required

4. For each of the following drugs, please **select the top 5 most important criteria** considered when **assessing whether to include the copy or replace the originator on your formulary** from the list below.

Please select the top 5 most important criteria **separately for the different drugs shown**.

Please assume each copy is approved for use in general in your country.

a. Please select the **most important** criterion **for the different drugs** shown.
b. Now please select the **second most important criterion for the different drugs** shown.
c. Now please select the **third most important criterion for the different drugs** shown.
d. Now please select the **fourth most important criterion for the different drugs** shown.
e. Now please select the **fifth most important criterion for the different drugs** shown.

Show item list and let the respondent select the top criterion for each type of drug on one screen

One screen per drug, order as in Q2

- Copy of the IV iron, iron carboxymaltose (Ferinject®)
- Copy of the immunomodulator, glatiramer acetate (Copaxone®)
- Copy of the TNF inhibitor, etanercept (Enbrel®)
- Copy of the painkiller, acetylsalicylic acid (Aspirin®)

Item list

Randomise items for each respondent, but maintain order for each ranking screen

- Price
- Supply reliability
- Clinical data specific to the copy drug (not extrapolated from originator) for safety and efficacy
- Head-to-head clinical study *vs* originator drug for safety and efficacy
- Mode of administration
- Non-active substance components (excipients)
- Manufacturing method
- Country manufactured in
- Guidance through national regulatory bodies
- Guidelines from indication-specific bodies (e.g. European Society of Endocrinology or similar)
- Licensed indication areas
- Mode of administration/galenic form/mode of preparation

5. Thinking of drug copies of originators, who **defines/would define whether it is substitutable** with its originator?

   Please select the appropriate answer from the options below.

One answer possible

- ❑ The **European Medicines Agency (EMA)** defines which drug copies are substitutable with originators
- ❑ **National bodies** define which drug copies are substitutable with originators
- ❑ **Local/regional bodies** define which drug copies are substitutable with originators
- ❑ It is up to **my hospital/institution** to define which drug copies are substitutable with originators
- ❑ Don't know

3. Awareness of EMA Guidance

6. **How familiar** are you personally with the following **approval application pathways** for drug copies from the European Medicines Agency (EMA)?

   Please indicate your level of familiarity of a scale from 0 to 10.
   0 = "not familiar at all" and 10 = "very familiar".

Scale from 0 to 10 for each pathway

- Generic approval pathway article 10(1)
- Hybrid approval pathway article 10(3)
- Biosimilar approval pathway article 10(4)

7. Based on your knowledge, what clinical data do copy drug manufacturers have to provide as part of each of the following **European Medicines Agency (EMA) approval application pathways**?

One screen per pathway

- Copy approved based on generic approval pathway Article 10(1)
- Copy approved based on hybrid approval pathway Article 10(3)
- Copy approved based on biosimilar approval pathway Article 10(4)

Multiple answers possible

- ❑ Clinical pharmacokinetics studies
- ❑ Full clinical trials in 1 indication
- ❑ Full clinical trials in all relevant indications
- ❑ Head-to-head clinical study *vs* originator

Single answers:

- ❑ No clinical data required
- ❑ Don't know

For each pathway with "don't know", ask:

8. You mentioned you do not know which data is required for the following pathway(s).

Based on your experience, what clinical data do **you expect** copy drug manufacturers have to provide as part of each of the following **European Medicines Agency (EMA) approval application pathways**?

Only show those answered with "don't know" in Q13. One pathway per screen

- Copy approved based on generic approval pathway Article 10(1)
- Copy approved based on hybrid approval pathway Article 10(3)
- Copy approved based on biosimilar approval pathway Article 10(4)

Multiple answers possible

❑ Clinical pharmacokinetics studies
❑ Full clinical trials in 1 indication
❑ Full clinical trials in all relevant indications
❑ Head-to-head clinical study *vs* originator

Single answers:

❑ No clinical data required

4. Hybrid Application Pathway Concept

9. You previously answered questions that referred to the European Medicines Agency (EMA) hybrid (Article 10(3)) approval application pathway for copy drugs.

To what extent would you expect a hybrid drug copy to be **identical, in terms of outcomes**, to its originator?

One answer possible

❑ No differences in outcomes possible *vs* originator
❑ Negligible differences in outcomes possible *vs* originator
❑ Notable differences in outcomes possible *vs* originator
❑ Don't know

10. Below you see a brief description of the EMA hybrid application pathway and hybrid medicines. Please take your time to read this description.

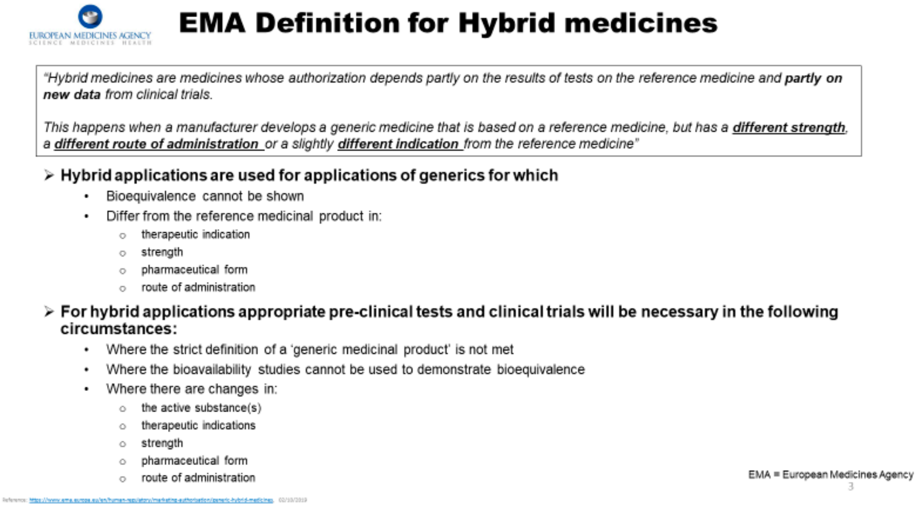

11. To what extent is this information **relevant for your daily practice** with regard to **listing** this type of hybrid (Article 10(3)) drug?

Please indicate how relevant this information is on a scale from 0 to 10.
0 = "not relevant at all" and 10 = "very relevant".

Scale from 0 to 10

12. In the case that a copy drug is approved via the hybrid (Article 10(3)) pathway, which of the following stakeholders do you believe would be **relevant in the decision to include it on the hospital formulary**?

Please select all relevant stakeholders.

Multiple answers possible

Randomise answer options

- ❑ Head hospital pharmacist
- ❑ Department-specific pharmacist (e.g. Hematology, Gastroenterology, etc.)
- ❑ *UK only:* High-cost-drugs pharmacist
- ❑ Prescribing clinicians
- ❑ Purchasing department
- ❑ Regional regulatory body
- ❑ *UK only:* National regulatory body/bodies (e.g. MHRA, NICE)
- ❑ *DE only:* National regulatory body/bodies (e.g. BfArM)
- ❑ *ES only:* National regulatory body/bodies (e.g. AEMPS)
- ❑ *IT only:* National regulatory body/bodies (e.g. AIFA)
- ❑ *FR only:* National regulatory body/bodies (e.g. ANSM, SMR, HAS)
- ❑ Hospital drugs/medicines committee
- ❑ Regional drugs/medicines committee
- ❑ Buying groups

13. And which would be the **3 most important** stakeholders in listing a copy drug approved along the hybrid (Article 10(3)) pathway on your formulary?

Show all stakeholders selected in previous question

14. This final question relates to the drug category of **nanomedicines**.

Which European Medicines Agency (EMA) pathway do you believe copies of nanomedicines should be approved through?

One answer possible

- ❑ Generic approval pathway Article 10(1)
- ❑ Hybrid approval pathway Article 10(3)
- ❑ Biosimilar approval pathway Article 10(4)

Thank you for taking part! This research was sponsored by Vifor Pharma.

Do you have any final comments on the survey topic or functionality (e.g. technical issues, wording, etc.)?

Open answer, do not force response

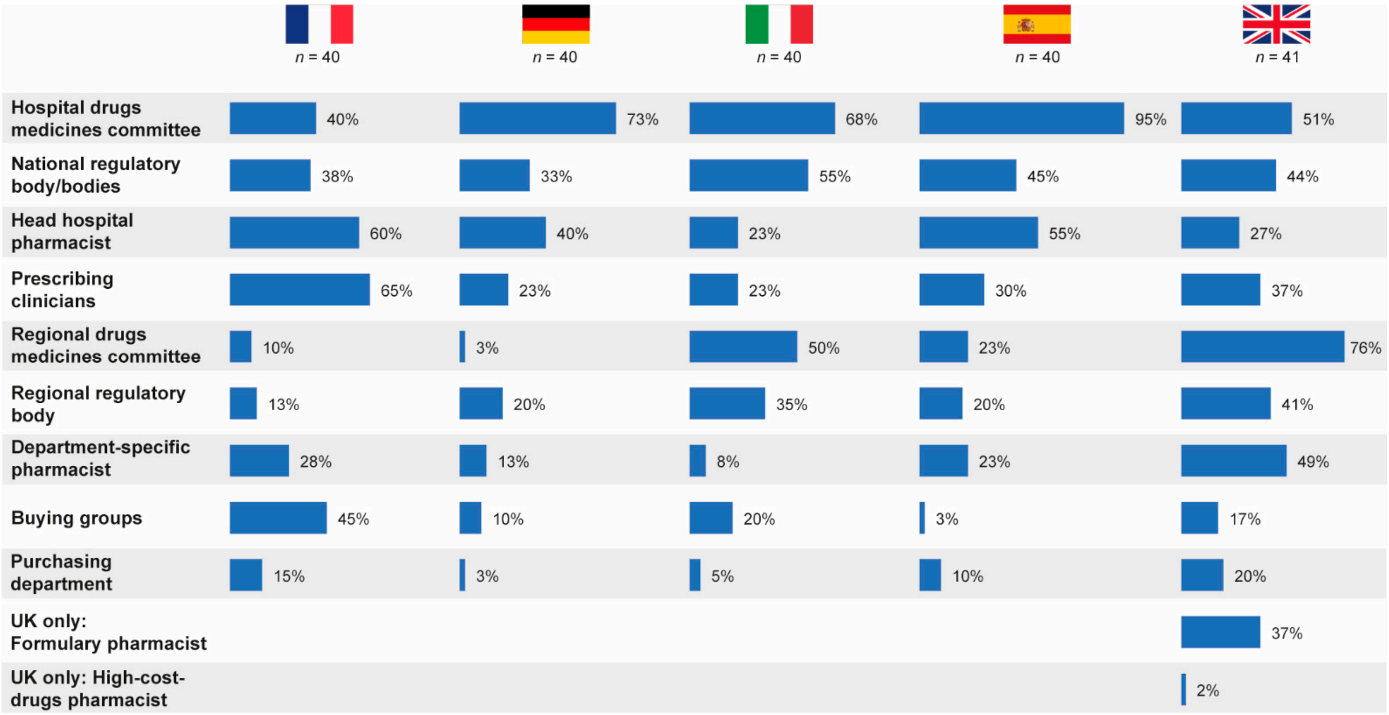

Figure S1. Stakeholders believed by pharmacists to be relevant in the decision to include a hybrid pathway-approved follow-on drug in the hospital formulary.

Source question: In the case that a copy drug is approved via the hybrid pathway, which of the following stakeholders do you believe would be relevant in the decision to include it on the hospital formulary? (Choose as many options as required.).
